# Roles of miRNAs in spinal cord injury and potential therapeutic interventions

**DOI:** 10.20517/2347-8659.2019.19

**Published:** 2019-10-17

**Authors:** Badria Almurshidi, Wayne Carver, Geoff Scott, Swapan K. Ray

**Affiliations:** 1 Department of Environmental Health Sciences, Arnold School of Public Health, CENR, University of South Carolina, Columbia, SC 29209, USA; 2 Department of Cell Biology and Anatomy, School of Medicine, University of South Carolina, Columbia, SC 29209, USA; 3 Department of Pathology, Microbiology, and Immunology, School of Medicine, University of South Carolina, Columbia, SC 29209, USA

**Keywords:** Spinal cord injury, miRNAs, astrogliosis, neuropathic pain, inflammation, apoptosis, functional recovery

## Abstract

Spinal cord injury (SCI) affects approximately 200,000 individuals per year worldwide. There are more than 27 million people worldwide living with long-term disability due to SCI. Historically, it was thought that the central nervous system (CNS) had little ability for regeneration; however, more recent studies have demonstrated potential for repair within the CNS. Because of this, there exists a renewed interest in the discovery of novel approaches to promote regeneration in the CNS including the spinal cord. It is important to know the roles of the microRNAs (miRNAs) in modulation of pathogenesis in SCI and the potentials of the miRNA-based clinical interventions for controlling post-injury symptoms and improving functional recovery. The miRNAs, which are non-coding RNAs with an average of 22 nucleotides in length, are post-transcriptional gene regulators that cause degradation of the target mRNAs and thus negatively control their translation. This review article focuses on current research related to miRNAs and their roles in modulating SCI symptoms, asserting that miRNAs contribute to critical post-SCI molecular processes including neuroplasticity, functional recovery, astrogliosis, neuropathic pain, inflammation, and apoptosis. In particular, miR-96 provides a promising therapeutic opportunity to improve the outcomes of clinical interventions, including the way SCI injuries are evaluated and treated.

## INTRODUCTION

Spinal cord injury (SCI) results from contusion/compression or transection of the spinal cord. SCI is a significant health issue with an estimate putting the number of people living with this neurological condition at more than 300,000 in the United States^[1]^. Because of the high number of casualties, SCI is also associated with various socio-economic challenges^[2]^. Hence, it is essential to understand the molecular mechanisms of pathogenesis in SCI in animal models and to elucidate novel therapeutic interventions for this devastating neurological condition^[3]^. The emergence of microRNAs (miRNAs) as potent regulators of gene expression at the post-transcriptional level has vast implications in many critical biological processes that include cell proliferation, differentiation, survival, and metabolism^[4]^. Studies indicate that miRNAs are currently attractive candidates as the upstream regulators of secondary injury progression in SCI because miRNAs are known to regulate entire sets of genes post-transcriptionally. Specific miRNAs (such as miR-96 and miR-544a) are potentially deregulated after SCI and the impact of this deregulation is an area of great interest^[5]^.

Bioinformatic analysis indicates that the potential targets of miRNAs altered after SCI include genes encoding components that are involved in inflammation, oxidative stress, and apoptosis, all of which are known to be crucial for progressive pathogenesis in SCI, suggesting that abnormal expression of miRNAs may contribute to the pathogenesis in SCI. Levels of expression of miRNAs were identified to be deregulated (decreased or increased) in SCI animals^[6]^. A later investigation showed dramatic decreases in the expression of miRNAs including miR-96 in SCI^[7]^. Upregulation of miR-96 is likely to promote cell proliferation^[8]^ and prevent neurodegeneration^[9]^ for contribution to functional neuroprotection in SCI. Because miRNAs highly decrease specific gene expression and deregulation of miRNAs does occur in SCI, the potential of particular miRNAs as therapeutic agents should now be explored for functional neuroprotection in SCI^[10]^. This review article mainly focuses on recent research related to changes in expression of miRNAs following induction of SCI and effects of modulation of levels of miRNAs on critical molecular processes including neuroplasticity, astrogliosis, neuropathic pain, inflammation, apoptosis, and functional recovery in SCI. The last section of this review article focuses explicitly on miR-96 as an emerging post-transcriptional regulator that has the potential to revolutionize SCI clinical interventions.

## SCI PATHOPHYSIOLOGY AND MIRNAS

**Figure 1.**
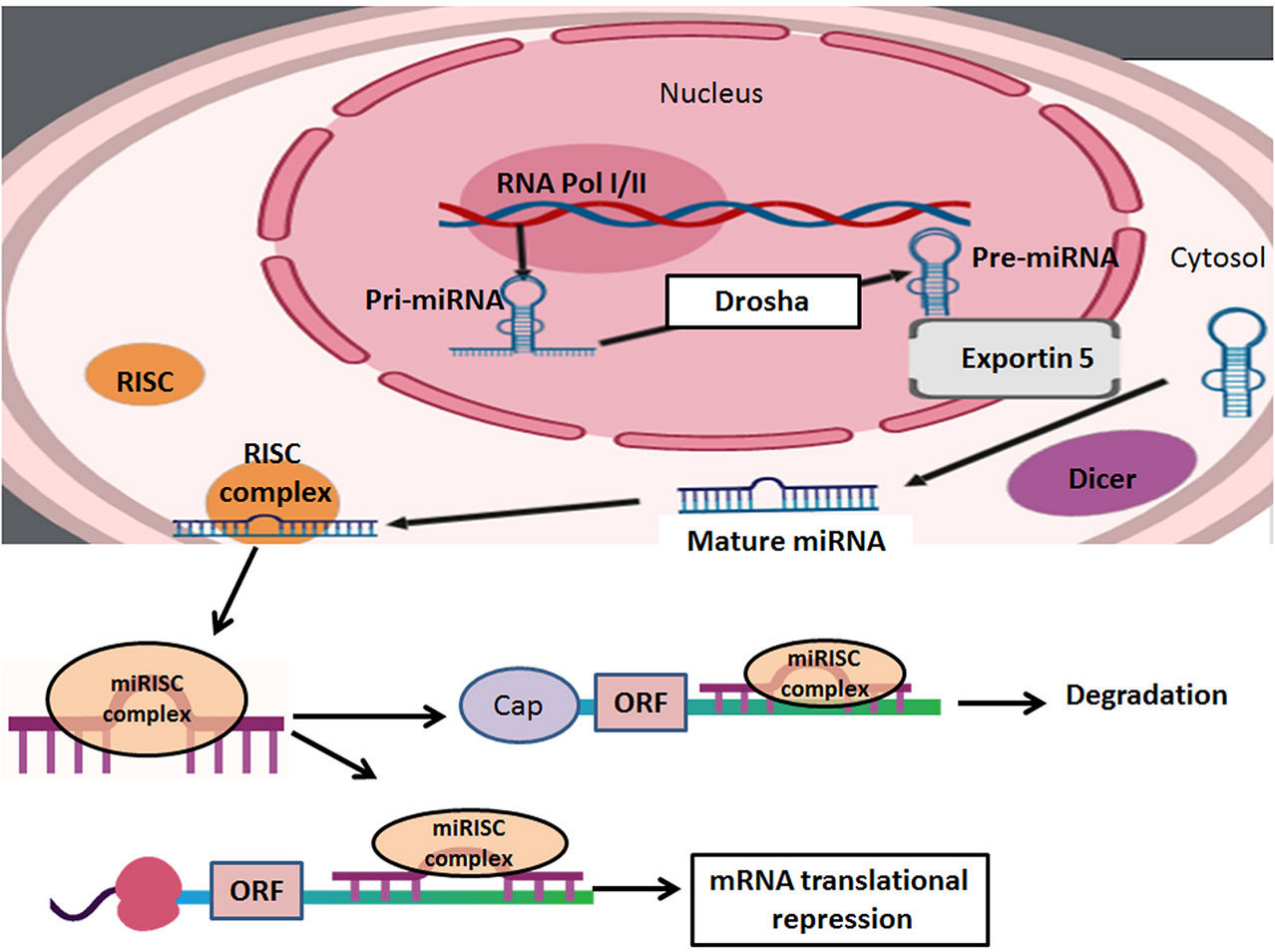
The biosynthesis of miRNAs begins in the nucleus. RNA polymerase (RNA pol) transcribes primary miRNAs, which consist of a poly-A tail and a 5’ cap. The multi-processor complex made up of double-stranded-RNA-binding protein and the RNase III enzyme Drosha to form pre-miRNAs. The Ran-GTP complex and karyopherin export pre-miRNAs into the cytoplasm. To finalize the process, the RNase III enzyme Dicer cleaves pre-miRNAs and triggers a further processing step by generating the miRNA. When miRNA-induced silencing complex (miRISC) is in the cytoplasm, miRNAs act on the target transcripts via complementary Watson-Crick base pairing to the corresponding miRNA response elements (MREs), which are usually present within the 3'-untranslated regions (3’UTRs) of target genes. Upon binding to MREs within 3’UTRs, miRNAs reduce protein outcome from the target transcripts due to translational repression and/or mRNA deadenylation and decay mechanisms

The occurrence of SCI is classified into two different stages: primary stage (a few moments following the initial injury) and secondary stage (hours, days, or weeks after the initial injury). Research shows that the first phase of SCI is the best predictor of future prognosis^[11,12]^. During the first phase, SCI is manifested with immediate changes in pathophysiology such as hemorrhage, Ca^2+^ overload, and activation of the Ca^2+^-dependent cysteine protease calpain causing necrotic and apoptotic neuronal death at the site of impact^[13]^. The secondary phase begins with molecular and physiological changes responsible for bleeding, loss of neurological functions, expansion of the lesion area, and overall amplification of the injury^[14,15]^. The secondary phase is also characterized by biochemical reactions, vascular alterations, inflammation, and edema^[16]^. The main pathological mechanisms responsible for the changes during the secondary phase are the depletion of energy, which is caused by ischemia and oxidative stress, neuroinflammation, and activation of calpains and caspases for cell death at the site of injury and the penumbra^[17]^. The combination of cellular and molecular modifications leads to various pathological events ranging from astrogliosis to apoptosis and tissue atrophy^[18]^. Understanding the pathological events is highly crucial for modulating progression of pathogenesis leading to activation of cysteine proteases in both acute and chronic SCI and for implementing therapeutic interventions^[19]^. It has been shown that attenuation of neuroinflammation and neurodegeneration with appropriate therapeutic interventions is essential for functional recovery in preclinical models of SCI^[20,21]^. A relatively novel therapeutic intervention evaluated for the treatment of SCI in preclinical models is the utilization of miRNAs^[22]^. The miRNAs are small non-coding RNA molecules that post-transcriptionally regulate gene expression by degrading and inhibiting translation of target mRNAs [Figure 1].

The biogenesis of miRNAs occurs from pre-miRNA precursors and miRNAs are present not only in body fluid (sputum, serum, and blood) but also in body tissues where they are associated with the regulation of expression of target mRNAs^[23]^. Different miRNAs are also found in the central nervous system (CNS) where they act as essential mediators of neurodevelopment in the brain and spinal cord in mammals. For example, specific miRNA expression (e.g., miR-17~92 clusters) controls neural growth as well as neurogenesis-gliogenesis switch in the developing CNS including spinal cord. Likewise, miRNAs target genes that are involved in the regulation of essential pathophysiological processes including apoptosis (miR-124 and miR-21), inflammation (miR-544a), and astrogliosis (miR-145 and miR-21) in the spinal cord^[24]^. The alterations in the expression of miRNAs following SCI can be categorized into three groups: (1) increased miRNAs; (2) decreased miRNAs; and (3) bidirectional (increased or decreased) miRNAs. The expression of specific miRNAs such as miR-146a and miR-129–2 is significantly influenced by the injury severity. The down regulation of specific miRNAs such as miR-219 and miR-124 is associated with the death of neural cells. On the other hand, the overexpression of specific miRNAs (e.g., miR-223) is due to infiltration of vascular and immune cells. Such infiltration of immune cells has been observed during the acute stage of SCI. Research has shown changes in miR-451 during the initial phase of SCI^[25]^. Other studies also indicate the important roles of miRNAs in regulating immune response and alleviation of inflammation following SCI. For example, miR-544a is down regulated after SCI while genes associated with inflammation (especially NEUROD4) are overexpressed^[26]^. These results suggest that miR-544a is essential in the repair process after SCI, although further research is required to confirm this assertion.

## POTENTIAL ROLES OF MIRNAS IN SCI

### miRNAs in astrogliosis

Astrogliosis or astrocytosis refers to the response of astrocytes in response to SCI. Astrogliosis occurs in the area close to the SCI and is a part of a complex multicellular response to SCI. Astrogliosis is characterized by functional, molecular, and morphological changes in astrocytes^[27]^. These changes usually occur within a few hours following the initial injury and evolve with time^[28]^. The process of astrogliosis is beneficial in the acute stages where it triggers the repair of the spine-blood barrier, cell regeneration, and prevention of inflammation. However, astrogliosis becomes detrimental in the later stages (4 to 6 weeks) following SCI when astrocytes change from hypertrophic to hyperplastic, producing glial scar with expression of chondroitin sulfate proteoglycans^[29]^.

Under specific pathological conditions, molecular signaling mechanisms associated with miRNAs affects the process of astrocyte proliferation and astrogliosis. For example, the addition of anti-miR-125b is linked with reduced glial cell proliferation and overall regulation of cell growth^[30,31]^. Similarly, overexpression of miR-145 following SCI has been shown to increase astrogliosis in astrocytes close to the injured area^[32]^. There was focus of another study, which sought to determine the regulating mechanism of miR-21 in regard to glial scars and astrocytic hypertrophy^[33]^. More specifically, these investigators carried out tests involving overexpression of miR-21 in mouse astrocytes and concluded that overexpression of miR-21 in astrocytes attenuated hypertrophic response to SCI but expression of the miR-21 sponge augmented hypertrophic phenotype, even in chronic phase of SCI. Other researchers have studied the role of miR-21 in the hypertrophy-hyperplasia shift. According to recent research, miR-21 suppresses the expression of the glial fibrillary acidic protein (GFAP) and vimentin (VIM) under the influence of bone morphogenic protein (BMP) receptors^[34]^. Activation of different BMP receptors results in differential expression of miR-21 in astrocytes and controls astrogliosis^[35,36]^. BMP receptor type 1a (BMPR1a) and BMPR1b exert opposite effects on reactive astrocytic hypertrophy. BMPR1b plays a role in glial scar progression in the chronic stages following SCI. These receptors exert opposite effects on expression of miR-21 in astrocytes. Activation of BMPT1a causes overexpression of miR-21 with a dramatic reduction in GFAP levels, limiting the detrimental effects of BMPR1b signaling on glial scar formation following SCI.

### miRNAs in apoptosis

**Figure 2.**
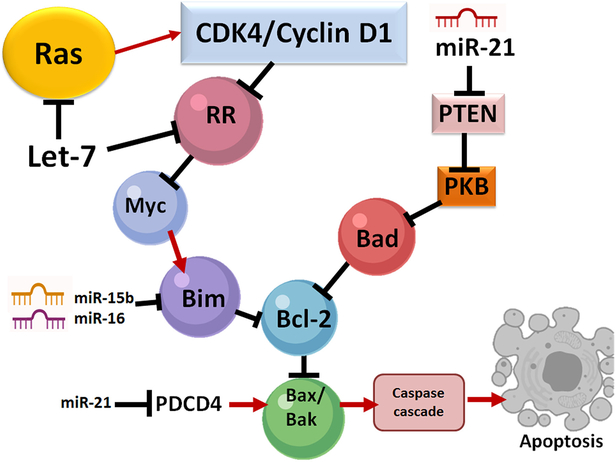
Regulation of apoptosis by miRNAs. All of miR-16, miR-15b, and miR-21 act on several molecular pathways, including Bak, Bcl-2, and Bax pathways during this process. miR-15b, miR-16, and Let-7 down regulate the expression of Bcl-2 (an anti-apoptotic protein) and trigger the release of cytochrome c (a pro-apoptotic factor). Down regulation of Bcl-2 reduces the mitochondrial membrane potential while cytosolic cytochrome c activates the caspase cascade, which subsequently leads to apoptosis. The role of miR-21 is two-fold: protection of neural cells and anhibition of apoptosis. Also, miR-21 down regultes PDCD4 to limit the activation of pro-apoptotic molecular pathways involving Bak and Bax. This, in turn, hinders apoptosis. Also, pro-apoptosis. PTEN is down regulated by miR-21, resulting in an anti-apoptotic effect

Some researchers studied the action of miRNAs in the context of apoptosis. For example, overexpression of miR-96–5p inhibits apoptosis but promotes migration and proliferation in MDA-MB-231 and MCF-7 cells^[37]^. In this study, these investigators overexpressed miR-96–5p by transfecting MDA-MB-231 and MCF-7 cells with a miR-96–5p mimic. Treatment and incubation of these cells were done for two days after which cells were double stained with Annexin V/propidium iodide. Flow cytometry was then used to quantify the apoptotic cells. The conceivable molecular events leading to induction of apoptosis in SCI are shown [Figure 2]. Upregulation of miR-96 may suppress programmed cell death protein 4 (PDCD4) and subsequently decrease apoptosis. It is known that reduction of miR-21 promotes apoptosis by inhibiting the expression of phosphatase and tensin homolog (PTEN) and PDCD4^[38]^. Down regulation of the pro-apoptotic factors PTEN and PDCD4 can increase expression of the cell survival factor Akt or protein kinase B, leading to a reduction in apoptosis in SCI. Studies indicated that specific miRNAs (e.g., miR-21, miR-7–1) could significantly enhance efficacy of promising therapeutic agents (e.g., estrogen receptor agonists) for functional protection of spinal cord motoneurons and this combination therapeutic strategy could be used in the future to attenuate apoptosis of motoneurons in SCI^[39,40]^.

### miRNAs in axon regeneration and remodeling

It has been demonstrated that exercise, which promotes spinal cord plasticity, results in the down regulation of miR-199a-3p and upregulation of miR-21^[41]^. Alteration in expression of these miRNAs modulates the mechanistic target of rapamycin (mTOR) and PTEN, which are postulated to underlie exercise-induced enhancement of neuronal regeneration in SCI^[42]^. In another study, it has been shown that upregulation of mTOR occurs by deleting the *PTEN* gene in mice, resulting in axon regeneration in the injured optic nerves^[43,44]^. Attenuation of axonal damage and neuronal death is highly crucial for recovery of locomotor function in preclinical models of SCI^[45]^. Animal model research also suggests that miR-210 carries regenerative properties to cause axon growth in the context of SCI^[46]^. Administration of miR-210 to SCI mice decreased the expressions of protein-tyrosine phosphate 1B and ephrin-A3 to contribute to spinal cord repair by promoting angiogenesis.

### miRNAs in neuronal cell cycle and functional recovery

There is evidence to show that miRNAs not only contribute to regeneration but also stimulate neuron growth and promote functional recovery^[47]^. Bioinformatic studies propose that miRNAs promote balance between the cell division cycle 42 gene and the brain-derived neurotrophic factor (*BDNF*) gene, both of which influence self-repair in SCI^[48]^. Studies show that miR-124 is associated with inhibition of neuronal apoptosis and improvement of motor scores in SCI, with the possibility of even restoring limb functionality after SCI^[49]^. Study suggests that miR-133b promotes neurite outgrowth via ERK1/2 and PI3K/Akt signaling pathway by RhoA suppression^[50]^. In terms of functional recovery, evidence shows that miR-133b has the ability to suppress the molecules that inhibit axon regrowth and thereby promote recovery of locomotor function after SCI^[51]^.

### miRNAs and neuropathic pain

A rich body of evidence has linked miRNAs to the regulation of SCI-related pain, both neuropathic and inflammatory^[52]^. According to researchers, the changes in miRNA expression induce increase in insulinlike growth factor-1 expression and down regulation of BDNF. This study concludes that the combination of these changes results in a decrease in inflammation and pain in SCI animals. Down regulation of miR-218 alleviates neuropathic pain by controlling the expression of cytokine signaling, which in turn inhibits the JAK/STAT3 pathway in SCI animals^[53]^. The expression of miR-124 mainly in neurons is found throughout the brain and spinal cord. A recent study has corroborated the sensitivity of miRNAs to SCI by showing that expression of miR-124 in neurons is significantly decreased within 7 days after SCI to show the severity of injury^[54]^. The dynamic changes in expression of miRNAs play multiple regulatory mechanisms that may be leveraged to reduce neuropathic pain and potentially shed new light on the progression of maladaptive plasticity in SCI^[55]^.

It has been demonstrated that miR-146a and miR-129–2 regulate pain during the early stages of SCI^[56]^. In this study, the investigators validated the expression of miRNAs by quantitative reverse transcription-polymerase chain reaction and *in situ* hybridization assays, revealing that SCI affected miRNA expression that persisted up to 14 days and expanded both anteriorly and caudally beyond the lesion site. It has been suggested that the effect of miRNAs on pain is not necessarily limited to the lesion site. It has been suggested that SCI induces changes in the expression of miRNAs in higher cortical structures, which control neuropathic and inflammatory pain, and miRNAs may serve as specific biomarkers for future targeted therapy of neuropathic and inflammatory pain conditions^[57]^.

## MOLECULAR INSIGHTS INTO ROLES OF MIR-96 IN SCI

### miR-96 in neuroprotective therapy in SCI

It is essential to mention that miR-96 is one of three miRNAs that make up the miR-183 cluster (the other two miRNAs of this cluster are miR-183 and miR-182)^[58]^. Recent studies confirm that expression of miR-96 is dramatically decreased after SCI, favoring induction of apoptosis due to increase in expression of its targets, which are pro-apoptotic proteins^[59]^. Another recent research showed that increase in expression of miR-96 suppressed microglia activation marker proteins and inhibited inflammatory cytokines such as tumor necrosis factor-α and interleukin-1β to promote recovery in SCI^[60]^. Additionally, it has been reported that miR-96–5p regulates cysteine transporters such as the excitatory amino acid (EAA) transporter 3/EAA carrier 1 (one of the amino acid carriers involved in neuronal glutathione synthesis), which produces neuroprotective benefits against oxidative stress^[61]^. According to these researchers, rhythmic diurnal fluctuations of glutathione levels occur when miR-96–5p is blocked indirectly influencing the neuroprotective effect^[62]^. The subsequent section of this article will describe recent research related to roles of miR-96 in inhibition of apoptosis, promotion of cell proliferation, and alteration of other molecular pathways.

### miR-96 in inhibition of apoptosis

Research shows that miR-96 may target and inhibit apoptotic factors at the protein and mRNA levels. For instance, miR-96 indirectly inhibits apoptosis through its effect on the forkhead transcription factor of the O class 1 (FOXOl) transcription factor^[63]^. Studies have shown that FOXO1 induces apoptosis via mitochondria-independent and mitochondria-dependent pathways. Also, miR-96–5p decreases the levels of caspase-9 (an important caspase in the mitochondrial pathway of apoptosis) by binding to the CASP9 3’-untranslated region (3’UTR)^[64]^. Overexpression of miR-96–5p is associated with inhibition of apoptosis^[65]^.

### miR-96 in cell proliferation

There is a growing interest in the study of miR-96, its effect on FOXO1 levels, and how this affects cell proliferation. For example, it has been shown that breast cancer causes upregulation of miR-96 by targeting protein tyrosine phosphatase, non-receptor type 9, this increases cell migration and proliferation and indirectly affects the pathophysiology mechanisms of breast carcinogenesis^[66]^. Studies on miR-96, specifically its role in hepatocarcinogenesis and treatment of hepatocellular carcinoma (HCC), have shown that miR-96 is significantly upregulated in HCC^[67]^. Studies also demonstrate that miR-96 targets the FOXO subfamily, specifically FOXO3a and FOXO1. Inhibiting miR-96 upregulates FOXO1 and FOXO3a expression, suppressing colony formation and cell proliferation in HCC^[68]^.

Studies have also shown that prostate cancer is associated with elevated expression of miR-96 and subsequent down regulation of FOXO1, a phenomenon that can be leveraged to control cell proliferation^[69]^. Other investigators studied the same phenomenon with a dataset containing non-malignant benign prostate tissue samples and prostate cancer tissue samples^[70]^. According to these researchers, overexpression of miR-96 decreases FOXO1 expression in both the non-malignant tissue samples and the prostate cancer tissue samples, even when the two samples are combined. Down regulation of FOXO3a and p27kip1 promotes axonal regeneration and proliferation of glial cells after SCI in rats^[71]^. An earlier investigation showed that down regulation of FOXO3a decreased p27kip1 at mRNA and protein levels after injury^[72]^. A recent study shows that miR-96 is highly essential for normal development of the auditory system, which is required for functional maturation in the peripheral and central auditory system^[73]^.

### miR-96 in regulation of FOXO pathway and other molecular pathways

**Figure 3.**
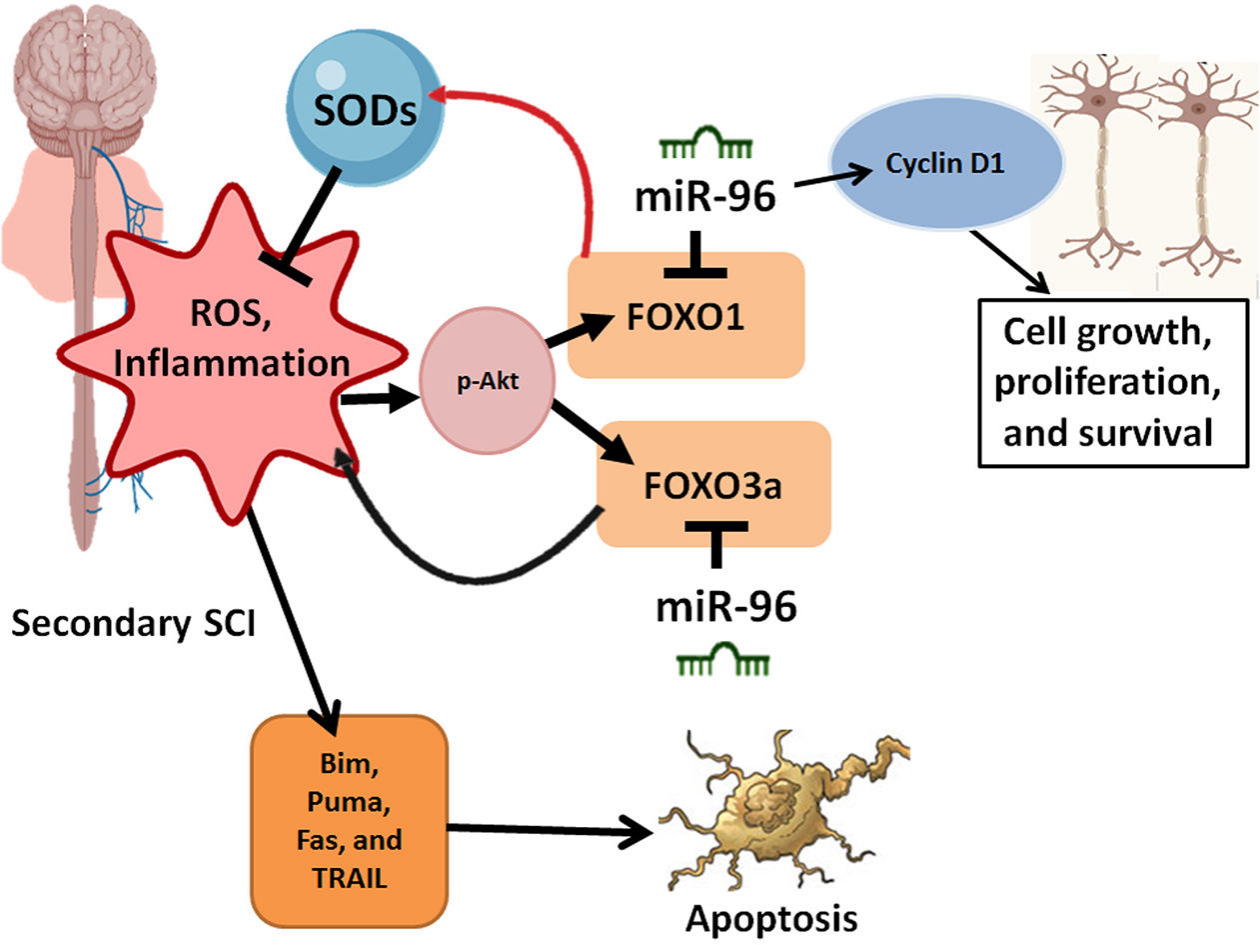
FOXOs facilitate cell cycle and metabolic regulation, which ultimately regulates apoptosis. At the beginning stage of spinal cord injury (SCI), FOXO1 activates the enzymes (e.g., superoxide dismutases or SODs) responsible for regulating oxidative stress from reactive oxygen species (ROS). With time FOXO1 induces autophagy, controls inflammation, and leads to cell cycle arrest. The role of miR-96 is most profound during the secondary stage of SCI, whereby it regulates FOXO proteins, including FOXO1 to enhances the survival of cells. FOXO3a is also down regulated by miR-96 affecting the expression of inflammatory pathways during SCI. This process also affects pro-apoptotic factors such as TRAIL, Fas, and Puma

FOXO pathways are of vital importance in regulating biological processes such as glucose metabolism, cellular proliferation, apoptosis, and repair of DNA damage^[74]^. FOXO transcription factors are also crucial in controlling neurodegenerative disorders through autophagy and apoptosis in the presence of oxidative stress^[75]^. Mounting evidence demonstrates that miR-96 plays a crucial role in regulating these FOXO pathways. For example, the expression of FOXO3a and FOXO1 is suppressed by miR-96, which controls the cell cycle, cell proliferation, and migration^[76]^. FOXO3a transcriptionally down regulates the level of p27kip1 (an important neurogenesis regulatory factor in mammals) positioning it as a candidate for controlling axonal regeneration after SCI. The role of miR-96 in regulating FOXO pathways has also been studied *in vitro*^[77]^, demonstrating that miR-96 binds to the FOXO1 3’UTR sequence lowering the transcript levels of FOXO1 and elevating cell growth [Figure 3].

Research shows that FOXO3a targets the cyclin-dependent kinase inhibitor p27kip1 and the pro-apoptotic molecule Bim, a phenomenon that triggers apoptosis^[78]^. Research has also shown that the effect of FOXO3a in regulating apoptosis is dependent on the expression of death receptor ligands such as the FasL^[79]^. There is evidence showing that miR-96 causes a reduction in the levels of both FOXO1 and FOXO3a for promoting cell proliferation^[80]^, an effect that can be further investigated for neuroprotection and regeneration in SCI.

## CONCLUSION

**Figure 4.**
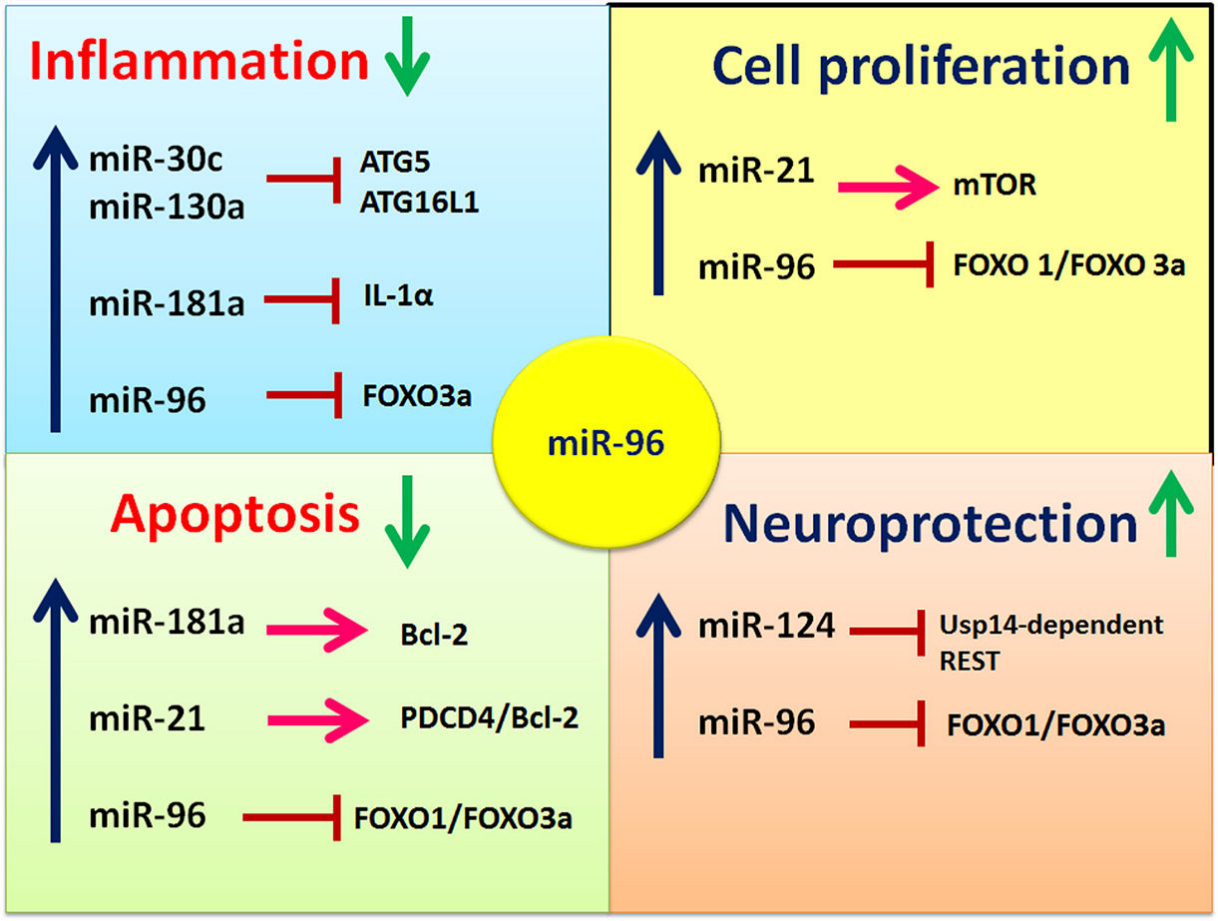
Summary of the roles of essential miRNAs including miR-96 in spinal cord injury (SCI). SCI affects the expression of miRNAs, which are known to regulate processes such as cell proliferation, inflammation, and apoptosis. Particularly, miR-96 is essential for neuroprotection as it induces multi-protective functions by targeting and down regulating FOXO pathways

Recent preclinical evidence makes it clear that miRNAs are useful therapeutic tools that modulate critical molecular processes and enhance functional recovery after SCI. In this article, we have discussed some examples, highlighting how the expression of miRNAs triggers complex interactions and changes at the cellular and protein levels. These changes affect SCI pathophysiology and have been identified as regulators and contributors to secondary injury. The assertions made in recent studies indicate that manipulating the expression of miRNAs may provide an opportunity for developing the improved therapeutic and clinical interventions for dealing with the devastating consequences of SCI [Figure 4]. In fact, it is essential to note that not all miRNAs affect SCI positively. However, many of the miRNAs discussed in this review article have been shown to contribute positively to the management of SCI. For example, miR-96 promotes axonal growth, cell regeneration, neuroplasticity, and facilitates functional recovery, although more research is needed in this regard.
